# Three-dimensional morphological evaluation of anatomical models for 101 primary maxillary central incisors

**DOI:** 10.1007/s40368-025-01058-x

**Published:** 2025-06-01

**Authors:** K. Li, A. Wen, J. Bai, M. Xu, T. Ma, D. Wang, Y. Zhao, B. Xia

**Affiliations:** 1 Department of Pediatric Dentistry, Peking University School and Hospital of Stomatology & National Engineering Research Center of Oral Biomaterials and Digital Medical Devices & Research Center of Engineering and Technology for Digital Dentistry of Ministry of Health & Beijing Key Laboratory of Digital Stomatology & National Clinical Research Center for Oral Diseases, Beijing, 100081 China; 2 Centre of Digital Dentistry, Peking University School and Hospital of Stomatology & National Center for Stomatology & National Clinical Research Center for Oral Diseases & National Engineering Research Center of Oral Biomaterials and Digital Medical Devices & Beijing Key Laboratory of Digital Stomatology & NHC Research Center of Engineering and Technology for Computerized Dentistry, Beijing, 100081 China; 3 Department of Oral Emergency, Peking University School and Hospital of Stomatology & National Engineering Research Center of Oral Biomaterials and Digital Medical Devices & Research Center of Engineering and Technology for Digital Dentistry of Ministry of Health & Beijing Key Laboratory of Digital Stomatology & National Clinical Research Center for Oral Diseases, Beijing, 100081 China

**Keywords:** Primary teeth, CBCT, Morphological study, 3D reconstruction, 3D average

## Abstract

**Purpose:**

Research on primary maxillary central incisors (PMCIs) remains limited, and their morphological variations have rarely been documented. This study aimed to reconstruct anatomy of PMCIs in children from Beijing (China) and to analyse their commonalities and variations.

**Methods:**

Employing a threshold-based semi-automated region segmentation method, anatomical models of 101 PMCIs were reconstructed from existing cone-beam computed tomography (CBCT) images. Models were classified according to the Vertucci classification. For each variant type, representative morphological parameters of the hard tissue, pulp chamber, and canals were measured. The type with the highest prevalence was selected as the main type and its average model was constructed, representing the most common characteristics of PMCIs.

**Results:**

All PMCIs had a single root, whereas novel canal variations were identified. The most common canal type was Type I (61.4%), followed by Type V (20.8%) and Type III (17.8%). Anatomical parameters of main-type PMCIs were as follows: hard-tissue length = 15.76 ± 0.89 mm; pulp chamber and canal length = 12.94 ± 1.15 mm; and apical labial curvature angle was 22.57°. Statistical analysis indicated no differences between left and right, and no sex-related differences (p > 0.05). Statistically significant differences between the main-type and other variants were noted for several pulp-chamber and canal measurements (p < 0.05), but not for hard-tissue measurements (p > 0.05). An average main-type model was constructed; its inner and outer profiles conformed to the general characteristics of main-type PMCIs.

**Conclusion:**

PMCI canal variations were more complex than previously recognised, with uniform hard-tissue anatomy. Furthermore, an average main-type model was constructed, as a potentially valuable tool for dental education.

## Introduction

Primary maxillary central incisors (PMCIs) are amongst the earliest teeth to erupt in the primary dentition. They play crucial roles in mastication, vocalization, space maintenance, and aesthetics. Dental caries and trauma pose major threats to PMCIs, not only compromising their hard-tissue integrity and function but also potentially causing pulp inflammation or necrosis in severe cases, which may progress to apical periodontitis. In some instances, infections of the oral-craniofacial complex may occur, whereas in others, the development of permanent successors may be disrupted (Dean 2021; Kim et al. 2023a; Torres-Ramos et al. 2020). A deep understanding of PMCI anatomical morphology and targeted training are essential for effective and successful clinical management.

Previous studies of primary teeth have demonstrated the complexity of their anatomical variations, which may be genetically or environmentally related and vary among different ethnicities (Lu et al. 2022; El-Messiry and Alaa 2021). Although primary anterior teeth are often described as being single rooted with single canals, other anatomical variations have been reported in the literature. For example, primary canines sometimes present with double-root variations, which often occur in children of African ancestry, and have been reported in Japanese and Indian children (Mochizuki et al. 2001; Orhan and Sari 2006). In addition, a single canal with bifurcation in the middle-third was found in 13% of primary mandibular incisors in Indian children (Gaurav et al. 2013). However, all current literature describes PMCIs as having single conical roots with a single root canal (Jung et al. 2012; Kim et al. 2023; Lyu et al. 2024; Torres-Ramos et al. 2020). Although children from diverse geographic regions, including Korea, China (Zhejiang), and Peru, were involved in these studies, these results do not fully reflect the effect of ethnicity on the morphology of PMCIs. These past studies included small sample sizes of 9–18 cases, raising concerns about undetected variations in PMCI morphology. Therefore, it is necessary to explore anatomical variations in PMCIs by including more samples.

Due to the physiological root resorption of primary teeth, collecting samples with intact roots for large in vitro anatomical studies is challenging, potentially limiting the discovery of morphological variations (Lyu et al. 2024; Torres-Ramos et al. 2020). A feasible solution is to conduct in vivo studies using cone-beam computed tomography (CBCT), which is widely used for diagnosing oral and maxillofacial pathologies in children (Jung et al. 2012; Patel and Horner 2009). It presents detailed three-dimensional (3D) anatomical information, with a definable field of view (Demiriz et al. 2018; Gaurav et al. 2013; Ozcan et al. 2016). The advent of digital technology has further enabled 3D modelling of teeth based on CT images, allowing researchers to observe tooth anatomy from a more stereoscopic perspective, and guide clinical operations with a more evidence-based rationale (Fu et al. 2020; Lahoud et al. 2022).

With limited access to extracted primary teeth, 3D printing can provide simulation substitutes for educational and research purposes; however, existing studies often focus on the batch printing of single ex vivo teeth models, which provides limited information (Liang et al. 2018; Moraes et al. 2019; Reymus et al. 2019).

More representative models can be constructed using a 3D averaging approach, which is performed on the basis of the statistical morphology analysis of a set of models in order to capture their common geometric characteristics (Besl and Mckay 1992; Gower 2001; Kim et al. 2023; Kuijpers et al. 2021; Wen et al. 2022). This is a relatively new digital technique that has not been commonly used in dental morphology studies. Kim et al. (2023) performed 3D averaging of the crowns and dental arches of permanent dentition. However, studies on the 3D averaging of anatomical tooth models with complete pulp chambers and canals are lacking.

The present study aimed to conduct a morphological analysis of PMCIs with a large convenience sample size; reconstruct their 3D anatomical models from CBCT scans of children in Beijing, China; and observe variations in their hard tissue, pulp chambers, and canals. Furthermore, the study aimed to create an averaged model representing the common characteristics of main-type PMCIs, to enhance the representativeness of 3D-printed tooth models.

## Materials and methods

### Data collection

Cone-beam computed tomography data for PMCIs were collected from CBCT scans of children aged 3–6 years who visited Peking University Hospital of Stomatology between January 2019 and April 2022. The scans were obtained for clinical diagnosis and treatment purposes unrelated to the present study. Qualified CBCT files were documented in the Digital Imaging and Communications in Medicine format. All procedures complied with the relevant laws and institutional guidelines. The requirement for informed consent was waived by our institution’s Ethics Committee, because the present study involved retrospective analysis of anonymised data and there was no additional risk to participants. The study was approved by the Ethics Committee (approval number: PKUSSIRB-202281147).

### Inclusion/exclusion criteria

The inclusion criteria included clear scans with full images of PMCIs showing complete apical development, no morphological abnormalities (for example, significant abnormalities in tooth size and shape, such as fused teeth, malformed cusps, dens invaginatus, and tooth dilacerations), no dental hard-tissue injury or caries lesion extending into the dentine layer radiographically, no internal or external root resorption, and no prior treatment. Scans with substantial motion artifacts were excluded.

### Sample size calculation

A pilot study was conducted to determine the appropriate sample size. The calculation was performed using PASS 2021 (NCSS LLC., Kaysville, UT, USA), with an alpha error of 0.05, an allowable error of ± 0.5 mm, and a known standard deviation of the length from the roof of pulp chamber to the root canal apex (this parameter was chosen to provide guidance for root canal treatment). The estimated sample size for each variant was 16.

### Radiographic techniques

This study was a retrospective study of previous data, and all CBCT scans were obtained for diagnose purposes unrelated to the study. Considering the requirements for imaging reconstruction accuracy, CBCT scans with the minimum slice thickness were selected. The 3D Accuitomo CBCT machine (J. Morita Manufacturing Corp., Kyoto, Japan) was used to obtain images with a voxel size of 0.125 mm. All images were acquired by licensed radiologists at 90 kV and 5.0 mA with an exposure time of 17.5 s.

### Sample model reconstruction and analysis

To ensure the accuracy of our results, all researchers have undergone necessary training processes to master the digital software involved and have passed the relevant examinations.

#### 3D modelling

**Fig. 1:**
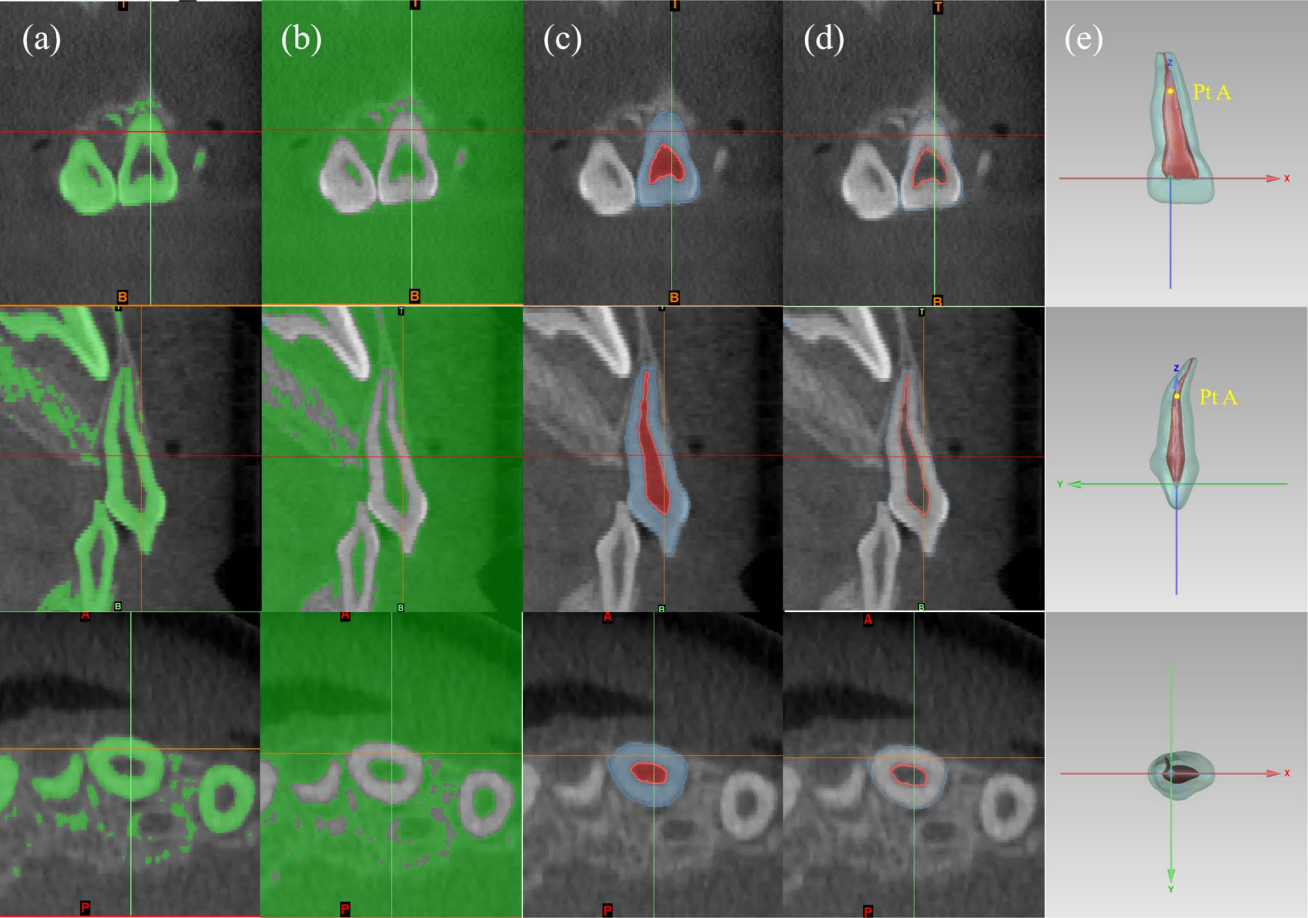
Segmentation and coordinate adjustment. **a** Threshold selection for hard tissue; **b** threshold selection for pulp chamber and canal; **c** extracted hard tissue (blue) and pulp chamber and canals (red); **d** profile lines for hard tissue (blue) and pulp chamber and canals (red), consistent with the original cone-beam computed tomography (CBCT) scan; **e** coordinate axes adjustment. Point A indicates the midpoint of the section at the apical labial curvature

The 3D morphology of the hard tissues and pulp cavity of PMCIs was obtained using Mimics 24.0 (Materialize Nv, Leuven, Belgium), based on a threshold-based semi-automatic segmentation method, which was then manually corrected. The PMCI models were reviewed by two paediatric dentists with over 20–30 years of clinical experience to ensure that they were consistent with the original CBCT, then exported in “.stl” format. In the case of differing opinions between the two experts, consensus was reached after discussion (Fig. 1).

#### Coordinate adjustment

The coordinate axis of the model was adjusted in Geomagic Control X (3D Systems Corp., Rock Hill, SC, USA), using the same criteria such that the connection line between the two pulp horns was aligned with the system’s x-axis; the line passing through the midpoint of the section at the apical labial curvature (point A) and perpendicular to the x-axis was aligned with the z-axis; and the line passing through the intersection of the x- and z-axes and perpendicular to these two lines was aligned with the y-axis (Fig. 1).

#### 2.5.3. Variation observation and model classification

A paediatric dentist observed the models from the coronal, sagittal, and vertical axes, classifying them by root numbers and root canal types according to the Vertucci classification (Vertucci 1984). The classifications were reviewed by a paediatric specialist with over 30 years of clinical experience and four other paediatric dentists. The observers were trained in morphological classification and passed the relevant examinations; thereafter, a pilot study was conducted to perform a standard consistency test on results of tooth-anatomy classifications by the observers. The Fleiss Kappa coefficient was 0.9 for inter-observer agreement, and the mean Cohen's Kappa was 0.96 for intra-observer reliability when reassessed after 1 month.

#### Measurements of hard-tissue features

The total length (L), crown height (H), crown width (W), and height-to-width ratio (R) of the crown were selected as representative morphological parameters of the hard tissues. A labial curvature was observed in the middle or apical third of all PMCI roots, dividing the length of the whole tooth into coronal (CL) and apical (AL) segments, bounded by point A (Fig. 1).

#### Measurements of pulp chamber and canals

**Fig. 2:**
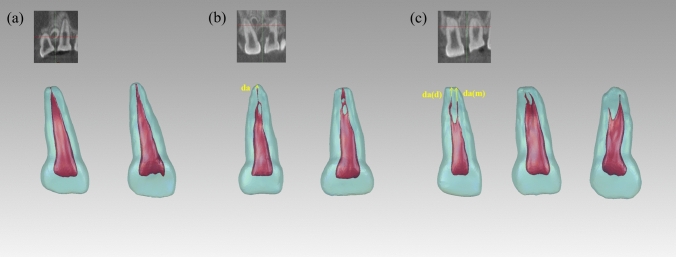
Variants of primary maxillary central incisors (PMCIs). **a** Type I: A single canal extending from the pulp chamber to the canal terminus, illustrated with a CBCT example and a distinct reconstructed tooth model. **b** Type III: A single canal bifurcating into two canals, which converge into one near the canal terminus; includes a CBCT example and a reconstructed tooth model (tooth labelled 61 was also Type III but excluded due to root resorption). **c** Type V: A single canal that bifurcates into two distinct canals before reaching the canal terminus; includes a CBCT example and a reconstructed tooth model. CBCT, cone-beam computed tomography

The volume (v) of the pulp chamber and canals was determined first. The entire length of the pulp chamber and canals (l) was then measured along the direction of each root canal, with multiple measurements taken if more than one canal was present. Considering the significant labial curvature of PMCIs, (l) was divided into a coronal segment (cl) and an apical segment (al). The ratio of the apical segment to the total length (p) was calculated. The distance (da) from the canal terminus to the anatomical apex of the root was also recorded (Fig. 2). The intersection angle between the coronal and apical sections of the canals was defined as the apical labial curvature angle (α), with an average angle recorded if multiple canals were present. A cross-section of the pulp cavity was made at half the root length, and its mesiodistal diameter (mdd), labio-palatal diameter (lpd), and mesio-distal-to-labio-palatal diameter ratio (r) were measured.

### Construction of the main-type average model

The variant with the highest prevalence amongst the PMCIs was defined as the main type, and an average model was created.

#### Template selection

The model with morphological parameters closest to the average was selected as the reference template for subsequent 3D averaging.

#### Model mirroring

If no discernible difference existed between the left and right sided teeth, all tooth models on the left side were mirrored using Geomagic Control X. A common average model was then calculated for both sides. For positional differences, separate average models were computed for each side.

#### Labelling of characteristics

**Fig. 3:**
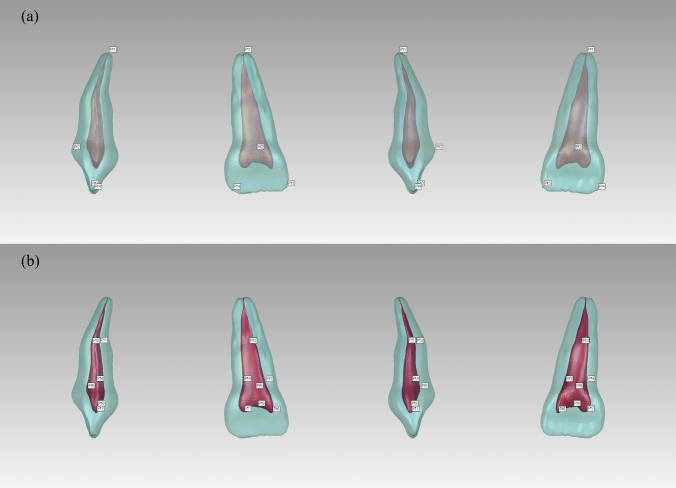
Alignment landmarks on hard tissue and pulp cavity. For hard tissue: Pt 1—apex of the root; Pt 2—most convex point on the cingulum; Pt 3—vertex of the mesial incisal angle; Pt 4—vertex of the distal incisal angle. For pulp cavity: Pt 1—most concave point on the labial side; Pt 2—most convex point on the palatal side; Pt 3—most mesial point at the root canal orifice level; Pt 4—most distal point at the root canal orifice level; Pt 5—most convex point on the lingual side of the pulp chamber; Pt 6—mesial pulp horn; Pt 7—distal pulp horn; Pt 8—centre point of the roof of the pulp chamber

Feature points on the external surface of the hard tissue and pulp cavity were selected as alignment landmarks. The coordinates (X, Y, and Z) of each landmark were manually identified three times. The mean value of these coordinates served as the final result, recorded in “.csv” format (Fig. 3).

#### Size normalization and alignment

The landmark coordinate set files of the selected reference template and remaining models were imported into MATLAB R2019b (MathWorks Inc., Natick, MA, USA). Using the Procrustes Analysis (PA) algorithm, size scaling coefficients and rotation matrices were determined for the remaining models based on the reference template (Gower 2001; Wen et al. 2022). These values were then applied to the corresponding models in Geomagic Control X to achieve size normalisation and alignment for all models.

#### Average model construction

Using the “Average” function in Geomagic Control X with the “Minimum Deviation” standard, the average models of the hard tissues as well as pulp chamber and canal were generated, respectively.

### Statistical analysis

All statistical analyses were performed using SPSS version 26.0 (IBM Corp., Armonk, NY, USA). The measurement indices were tested for normality using the Kolmogorov–Smirnov (K–S) test and for homogeneity of variance, with position (left and right) and sex as categorical variables. For indices that were normally distributed and homogeneous, comparisons were made using an independent sample t-test. For indices that did not conform to a normal distribution, the Mann–Whitney U test was employed. Subsequently, the same tests were conducted using the type of variation as a categorical variable. The significance level (alpha level) was set at 0.05.

## Results

### Morphological observations

#### Morphology of the hard tissue

In total, 65 files from 45 boys and 20 girls (mean age, of 4.2 years [range: 3–6 years]), were included. In total, 101 PMCIs were analysed, 50 from the left side and 51 from the right.

**Fig. 4:**
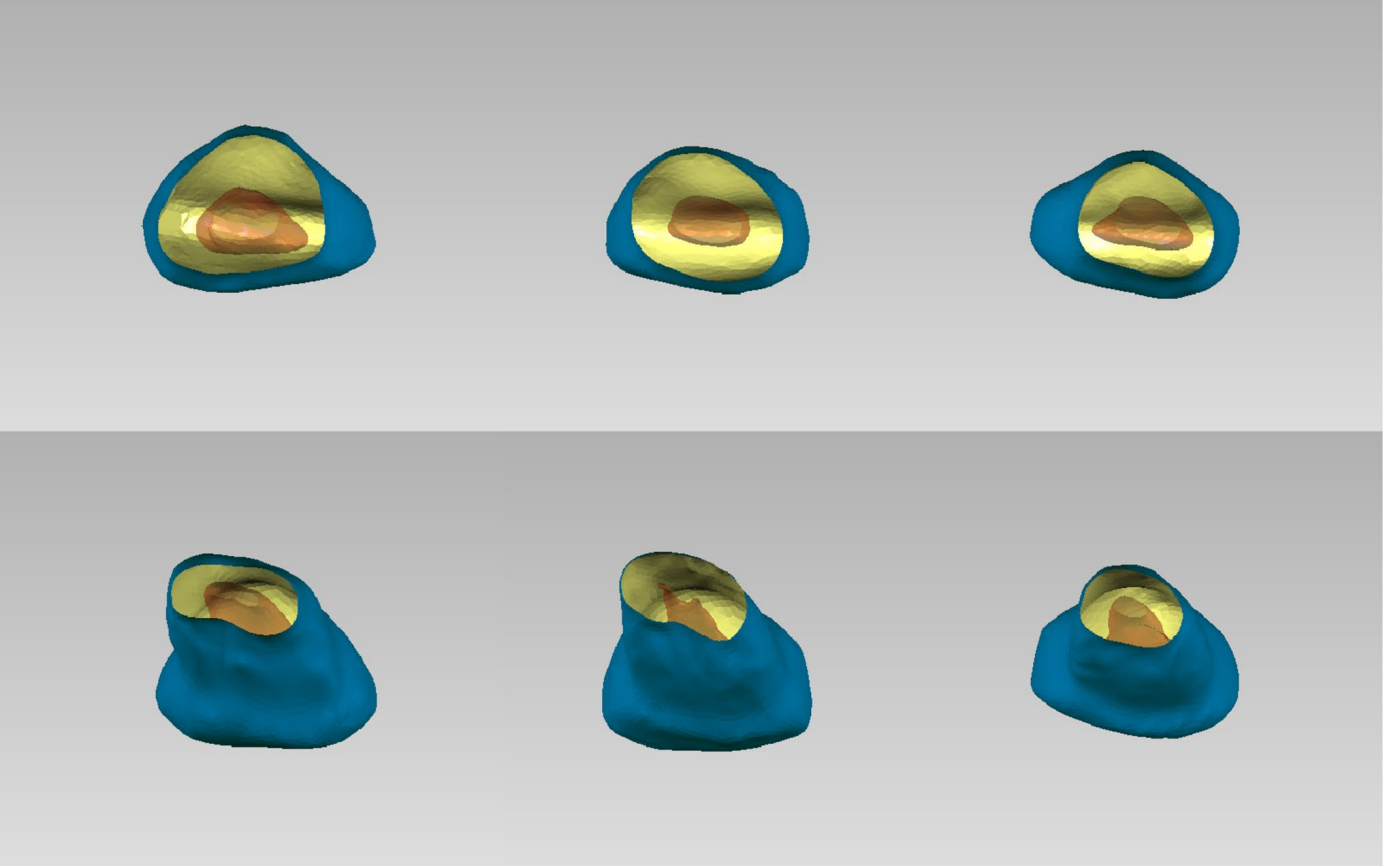
Cross-sectional variations for primary maxillary central incisors. Partial representation illustrating the different cross-sectional shapes of primary maxillary central incisors

The common hard tissue features of the 101 PMCIs were as follows: the crowns were spade-shaped, with a width greater than their length, and featured distinct cingula and dental cervixes. The mesio-incisal angles were generally acute (approximately 90°), whereas the disto-incisal angles were obtuse. All PMCIs had one root; therefore, no classification was performed accordingly. The roots were distally inclined below the dental cervix and labially inclined in the apical third. Throughout the entire length of the tooth, the mesiodistal diameter was greater than the labio-palatal diameter. The cross-sections of the roots were round-triangular or oval in the coronal third of the roots and transitioned to oval, flatter round-triangular, or kidney-shaped in the middle and apical thirds of the roots (Figs. 2, 4).

#### Morphology of the pulp chamber and canals

The shape of the pulp chambers and canals adapted to the hard tissue, although several variations were observed. The pulp chamber was flat, with mesio-distal diameters considerably wider than the labio-palatal diameters. All models were distally inclined from the root canal orifice to the apex, with a significant labial curvature in the middle or apical third. According to the Vertucci classification, 61.4% of the PMCIs exhibited Type I canals, 17.8% had Type III canals, and 20.8% had Type V canals (Fig. 2). The mesio-distal diameters were consistently larger than the labio-palatal diameters in all sections of all root canal types. Cross-sections of the single canals of Type I PMCIs were predominantly oval. The cross-sections in the single-canal parts of Type III were oval, gradually flattening into ribbon- or dumbbell-shaped sections before transitioning into two oval canals and converging to a single canal near the canal terminus. The two Type III root canals were close to each other, and fusion often occurred. The canals of Type V were wider and flatter compared to the other types. The cross-sections generally showed an oval or ribbon shape in the single canal parts, transitioning to dumbbell-shaped canals before splitting into two oval canals, which were spaced apart. No intercanal anastomoses were observed (Fig. 4). In Type III and Type V PMCIs, furcations were located in the middle or apical third of the root, where the canals separated mesiodistally. Most Type I and III root canals remained identifiable until reaching the apical foramen; however, most Type V canals were often too small to be identified before reaching the anatomical apex of the root (Fig. 2).

Based on these results, all PMCIs were classified into three types according to the Vertucci classification of their root canals, and those with Type I root canals were defined as the main-type PMCIs.

### Sample measurements

The mean hard-tissue length of the main type PMCIs was 15.76 ± 0.89 mm, with a mean crown height of 5.69 ± 0.39 mm, a mean crown width of 6.86 ± 0.30 mm, and a mean height-to-width ratio of 0.84 ± 0.07. There were no significant differences based on side (left or right) or sex in these measurements (*p* > 0.05; Table 1). The hard-tissue parameters for the other two types were statistically different from those of the Type I PMCIs (*p* > 0.05; Tables 2 and 3).

**Table 1:** Measurements of main-type (Type I) primary maxillary central incisors

Type I	Mean	Standard Deviation	Max	Min	Median	Average model
L (mm)	15.76	0.89	17.32	13.4	15.72	15.78
UL (mm)	11.03	0.51	11.75	9.33	11.1	11.06
LL (mm)	4.73	0.48	5.65	3.97	4.66	4.72
H (mm)	5.69	0.39	6.38	4.84	5.78	5.72
W (mm)	6.86	0.3	7.42	6.3	6.86	6.91
R	0.84	0.07	0.97	0.71	0.83	0.83
v (mm^3^)	20.44	5.75	35.31	8.19	18.45	20.55
l (mm)	12.94	1.15	16.13	10.39	12.9	12.93
ul (mm)	8.37	0.7	9.68	6.95	8.34	8.37
ll (mm)	4.57	0.68	6.46	3.06	4.64	4.56
p	0.35	0.03	0.43	0.27	0.36	0.35
da (mm)	0.15	0.27	0.91	0	0	0.16
α (°)	23.61	4.78	35.6	13.67	23.26	23.52
mdd (mm)	1.52	0.31	2.23	0.87	1.51	1.58
lpd (mm)	0.8	0.21	1.4	0.39	0.79	0.82
r	1.97	0.48	3.42	1.29	1.89	1.92

L, length of the whole tooth; CL, length of the whole tooth (coronal part); AL, length of the whole tooth (apical part); H, the height of the crown; W, the width of the crown; R, the ratio of crown height to width; v, volume of the pulp; l, pulp length; cl, pulp length (coronal part); al, pulp length (apical part); p, the ratio of the length of the apical segment of pulp to the total pulp length; da, the distance from the apex of the pulp to the hard tissue; α, angle of the apical labial curvature; mdd, the mesiodistal diameter; lpd, the labiopalatal diameter; r, cross-sectional diameter ratio

**Table 2:** Measurements of Type III primary maxillary central incisors

Type III	Mean	Standard Deviation	Max	Min	Median
L (mm)	15.62	1.01	17.23	13.44	15.64
UL (mm)	11.02	0.61	12.06	9.53	10.95
LL (mm)	4.6	0.47	5.49	3.83	4.63
H (mm)	5.61	0.31	6.1	4.95	5.64
W (mm)	6.9	0.29	7.35	6.26	6.85
R	0.82	0.05	0.94	0.72	0.83
v (mm)	17.14	4.17	27.9	10.59	16.01
l (m) (mm)	12.51	0.85	13.52	10.69	12.67
ul (m) (mm)	8.16	0.6	9.27	6.98	8.18
ll (m) (mm)	4.35	0.6	5.32	3.16	4.4
p (m)	0.35	0.04	0.41	0.29	0.35
l (d) (mm)	12.42	0.83	13.4	10.4	12.57
ul (d) (mm)	8.08	0.68	9.27	6.51	8.21
ll (d) (mm)	4.34	0.4	4.93	3.6	4.39
p (d)	0.35	0.03	0.4	0.3	0.35
da (mm)	0.22	0.27	0.7	0	0
α (°)	23.35	3.23	29.03	17.63	23.42
mdd (mm)	1.5	0.39	2.31	0.75	1.42
lpd (mm)	0.62	0.22	1.01	0.29	0.65
r	2.46	1.01	5.05	0.48	2.21

L, length of the whole tooth; CL, length of the whole tooth (coronal part); AL, length of the whole tooth (apical part); H, the height of the crown; W, the width of the crown; R, the ratio of crown height to width; v, volume of the pulp; l, pulp length; cl, pulp length (coronal part); al, pulp length (apical part); p, the ratio of the length of the apical segment of pulp to the total pulp length; da, the distance from the apex of the pulp to the hard tissue; α, angle of the apical labial curvature; mdd, the mesiodistal diameter; lpd, the labiopalatal diameter; r, cross-sectional diameter ratio

“(m)” indicates measurement data obtained along the mesial root canal; “(d)” indicates measurement data obtained along the distal canal

**Table 3:** Measurements of Type V primary maxillary central incisors

Type V	Mean	Standard Deviation	Max	Min	Median
L (mm)	15.64	0.62	16.92	14.64	15.54
UL (mm)	11.04	0.37	11.6	10.5	11.01
LL (mm)	4.6	0.35	5.32	4.14	4.53
H (mm)	5.61	0.52	6.58	4.79	5.58
W (mm)	7.13	0.5	8.08	6.34	7.14
R	0.79	0.05	0.87	0.72	0.81
v (mm)	16.77	4.55	24.92	10.14	17.89
l (m) (mm)	10.78	0.86	12.28	8.68	10.76
ul (m) (mm)	8.05	0.49	8.95	7.03	7.93
ll (m) (mm)	2.73	0.74	3.9	1.59	2.67
p (m)	0.25	0.05	0.33	0.16	0.25
da (m) (mm)	1.55	0.51	2.17	0.63	1.7
l (d) (mm)	11.78	0.65	12.9	10.35	11.84
ul (d) (mm)	8.07	0.45	8.97	7.41	7.97
ll (d) (mm)	3.71	0.63	5.2	2.51	3.62
p (d)	0.32	0.04	0.41	0.24	0.31
da (d) (mm)	0.65	0.47	1.54	0	0.52
α (°)	23.63	5.66	36.1	14.3	23.35
mdd (mm)	2.03	0.32	2.49	1.39	2.14
lpd (mm)	0.64	0.21	1.06	0.25	0.68
r	3.49	1.01	6.18	2.19	3.19

L, length of the whole tooth; CL, length of the whole tooth (coronal part); AL, length of the whole tooth (apical part); H, the height of the crown; W, the width of the crown; R, the ratio of crown height to width; v, volume of the pulp; l, pulp length; cl, pulp length (coronal part); al, pulp length (apical part); p, the ratio of the length of the apical segment of pulp to the total pulp length; da, the distance from the apex of the pulp to the hard tissue; α, angle of the apical labial curvature; mdd, the mesiodistal diameter; lpd, the labiopalatal diameter; r, cross-sectional diameter ratio

“(m)” indicates measurement data obtained along the mesial root canal; “(d)” indicates measurement data obtained along the distal canal

The mean volume of the pulp chambers and canals in the main-type PMCIs was 20.44 ± 5.75 mm^3^, and the mean length was 12.94 ± 1.15 mm. The average ratio of the length of the apical segment to the total length of the pulp chamber and canal was 0.35 ± 0.03, with the average angle of the apical labial curvature was 23.61 ± 4.78°, and the cross-sectional diameter ratio at half the root length was 1.97 ± 0.48. Statistical analyses indicated no significant differences based on location (left or right) or sex in these measurements (*p* > 0.05; Table 1). The apical labial curvature angle of Types III and V PMCIs was not significantly different from that of the main type (*p* > 0.05). However, the mean pulp cavity volumes of Types III and V were significantly smaller than that of the main-type. The labio-palatal diameter at half the root length in Types III and V was significantly smaller than that of the main type, and the mesiodistal diameter in Type V was significantly larger than that of the main type (*p* < 0.05), whereas no difference was observed between the mesiodistal diameters of Types III and I (*p* > 0.05). The cross-sectional diameter ratios of Types III and V were significantly higher than those of the main type (*p* < 0.05). All vertical length related measurements of the pulp cavity showed no difference between Types III and I (*p* > 0.05). Compared to Type I, Type V was significantly smaller in all vertical length-related measurements of the pulp cavity (*p* < 0.05), except for the coronal segment length, which was not different from that of the main type (Tables 2, 3).

It is noteworthy that all measurements of PMCIs followed a normal distribution, except for the distance (da) from the canal terminus to the anatomical apex of Types I and III. These deviations from were due to most root canals reaching the apical foramen in both types, resulting in a “da” measurement of 0. There was no difference in “da” between Types I and III (*p* > 0.05). However, the “da” of both canals in Type V was significantly greater than that in the main type (*p* < 0.05; Tables 1, 2, 3).

### Construction of the main-type average model

**Fig. 5:**
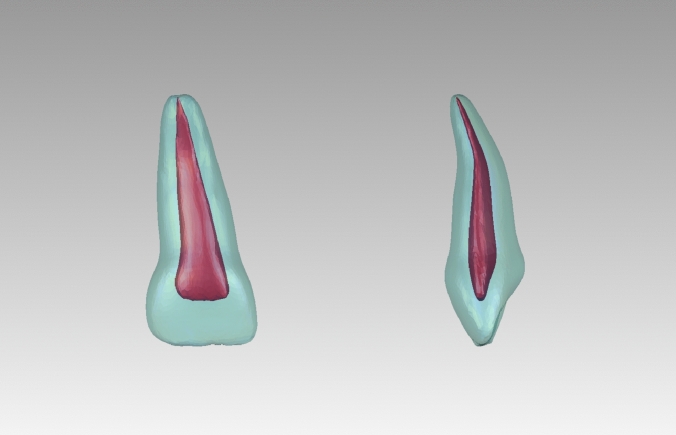
3D average model for main-type primary maxillary central incisors

A 3D average model of 62 main-type PMCIs was generated. The morphological characteristics of the averaged model were consistent with those of most main-type PMCIs, and its external and internal structures were adapted to each other (Fig. 5). All morphological parameters of the average model were within the 95% confidence intervals for the mean of the main type (Table 1).

### Current applications

**Fig. 6:**
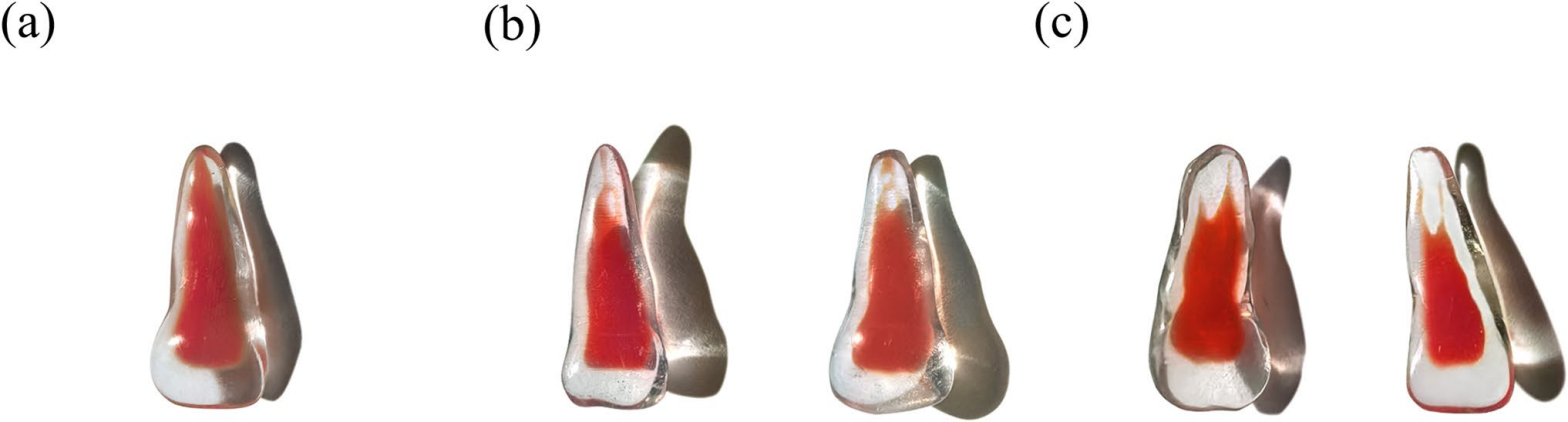
3D printed replicas of average main-type model and variations. (**a**) Average main-type model of primary maxillary central incisors, batch printed for preclinical practice and experimental use; (**b**) Type III variants presented as teaching cases; (**c**) Type V variants presented as teaching cases

The artificial PMCI models were constructed using high-precision 3D printers. The average main-type model was batch printed for preclinical practice and experimental use, whereas other variants were presented as teaching cases (Fig. 6).

## Discussion

Effective clinical practice in paediatric dentistry requires a profound understanding of tooth anatomy. Previous studies have reported the anatomical morphology of PMCIs in children of different ethnicities, but no variants other than Vertucci type I were identified. (Gaurav et al. 2013; Jung et al. 2012; Lyu et al. 2024; Torres-Ramos et al. 2020). This may be attributed to the relatively small sample sizes in these studies, potentially leading to possible variations being overlooked. However, these findings might suggest that Vertucci Type I is generally predominant in PMCIs across different populations. The present study, with a relatively large sample size of 101 cases, identified three root canal variants in PMCIs in children from Beijing, China, confirming that a significant proportion (38.6%) of canals bifurcate mesio-distally and providing detailed descriptions of PMCI anatomy. The novel findings of this study may be associated with the inclusion of a larger sample size, and might reflect certain ethnic-specific characteristics. Whether Type III and Type V PMCIs occur in other populations requires further investigation.

PMCIs with three different canal types exhibited no differences in the outlines and measurement results of their hard tissues. However, the canal cross-sections of Types III and V canals were flatter than those of Type I at half the root length, suggesting that during root canal therapy (RCT), the occurrence of two canals should be considered if a flatter root canal is present. In such cases, cautious exploration should be conducted in the mesiodistal direction, particularly in the middle and apical thirds of the canal.

Compared with Type I (the main-type), diameters of the root canals in Types III and V were relatively smaller below the bifurcation, with variable changes in canal direction, potentially making RCT more difficult. Most of the canal termini in Types I and III reached or were close to the anatomical apex; however, the distance between the canal terminus and the anatomical apex in Type V was significantly greater than in the other two types, putatively due to the small root canal diameter in the apical third. If the measurable working length is significantly shorter than the normal range (15.76 + 0.89 mm), chemical preparation should be enhanced appropriately (Elfarraj et al. 2024; Moradi and Haghgoo 2018; Ozdemir et al. 2012). Additionally, the labial curvature (23.61 ± 4.78° in our study) in the apical third of all PMCIs should be considered during treatment.

Previous studies on PMCIs have also reported various morphological parameters of Vertucci Type I PMCIs. For instance, Jung et al. (2012) conducted a CBCT study on PMCIs in Korean children and recorded an overall tooth length of 15.95 mm (crown length: 5.43 ± 0.51 mm; root length: 10.52 ± 0.62 mm) and a labial curvature angle of 26.3 ± 6.26°; these findings are comparable to the findings of the present study. In addition, the mean root canal length of Type I PMCIs in our study was 9.92 mm. Lyu et al. (2024) reported a similar but slightly shorter root canal length of 9.43 ± 1.35 mm in PMCIs from children in Zhejiang, China. Torres-Ramos et al. (2020) observed longer root canals in Peruvian children, with most measurements ranging between 10 and 11 mm. In contrast, Gaurav et al. (2013) documented a relatively shorter root canal length of 8.14 ± 0.93 mm in Indian children. These discrepancies in measurements may be attributed to ethnic variations or methodological differences.

In the present study, an averaged model was constructed for the main-type PMCIs, which had representative morphology, with each measurement close to the statistical mean, aiding clinicians in grasping the common characteristics of main-type PMCIs. Considering the rarity of intact extracted primary teeth, batch printing of ‘averaged teeth’ could help address the shortage of suitable teeth for preclinical practice and also be used to validate the efficiency of RCT instrumentation (Liang et al. 2018; Moraes et al. 2019; Reymus et al. 2019). The coronal data of this model can serve as a reference for restoration of carious lesions, as well as for 3D printing preformed crowns or strip crowns (Kim et al. 2023a; Waggoner 2015).

The present study has some limitations. As intact extracted PMCIs are difficult to obtain, high-precision in vitro methods were excluded in favour of CBCT (Fumes et al. 2014; Lyu et al. 2024; Orhan et al. 2015; Torres-Ramos et al. 2020), which ensured an adequate sample size whilst potentially resulting in subtle anatomical variations not being recognised. The CBCT in this study was obtained with a 0.125-mm slice thickness. Its reliability in identifying root canal numbers has previously been demonstrated using modified canal staining and clearing techniques as the gold standard (Neelakantan et al. 2010). Therefore, the conclusions of this study can provide a persuasive basis for root canal therapy. However, the CBCT resolution remains relatively low compared to that of micro-CT, potentially resulting in subtle anatomies being unrecognized. Previous studies have shown that CBCT is inferior to micro-CT in presenting complex root canal morphologies such as isthmuses, accessory canals, and apical deltas (Zhang et al. 2017). This is indeed a limitation of this study. Future research employing higher-resolution imaging modalities on well-preserved ex vivo PMCI specimens would be valuable for investigating these intricate anatomical structures in greater detail.

In addition, the CBCT scans in the present study were all obtained from children in Beijing, China; thus, the observed root canal variations and their incidence could be influenced by ethnicity. Future studies should explore PMCI variants in other populations.

## Conclusion

The present study identified novel root canal variations in PMCIs, establishing a foundation for more accurate treatments, including coronal restorations and endodontic procedures. Additionally, a representative 3D average model of the most common PMCI type was constructed using advanced digital techniques, offering a valuable tool for preclinical training and dental experiments, particularly for cases with limited access to extracted teeth. In the future, the digital construction of anatomical tooth models may serve clinical practice at a higher level.

## Data Availability

The data that support the findings of this study are available from the corresponding author upon reasonable request.
